# Magno- and Parvocellular Contrast Responses in Varying Degrees of Autistic Trait

**DOI:** 10.1371/journal.pone.0066797

**Published:** 2013-06-18

**Authors:** Brianna L. Jackson, Ellie M. Blackwood, Julieanne Blum, Sean P. Carruthers, Sabrina Nemorin, Brett A. Pryor, Shannon D. Sceneay, Stephanie Bevan, David P. Crewther

**Affiliations:** Centre for Human Psychopharmacology, Swinburne University of Technology, Melbourne, Victoria, Australia; University College London, United Kingdom

## Abstract

Autistic tendency has been associated with altered visual perception, especially impaired visual motion sensitivity and global/local integration, as well as enhanced visual search and local shape recognition. However, the neurophysiological mechanisms underlying these abnormalities remain poorly defined. The current study recruited 29 young adults displaying low, middle or high autistic trait as measured by Baron-Cohen's Autism spectrum Quotient (AQ), and measured motion coherence thresholds psychophysically, with manipulation of dot lifetime and stimulus contrast, as well as nonlinear cortical visual evoked potentials (VEPs) over a range of temporal luminance contrast levels from 10% to 95%. Contrast response functions extracted from the major first order and second order Wiener kernel peaks of the VEPs showed consistent variation with AQ group, and Naka-Rushton fits enabled contrast gain and semi-saturation contrasts to be elicited for each peak. A short latency second order response (previously associated with magnocellular processing) with high contrast gain and a saturating contrast response function showed higher amplitude for the High AQ (compared with Mid and Low groups) indicating poorer neural recovery after rapid stimulation. A non-linearity evoked at longer interaction times (previously associated with parvocellular processing) with no evidence of contrast saturation and lower contrast gain showed no difference between autism quotient groups across the full range of stimulus contrasts. In addition, the short latency first order response and a small, early second order second slice response showed gain and semi-saturation parameters indicative of magnocellular origin, while the longer latency first order response probably reflects a mixture of inputs (including feedback from higher cortical areas). Significant motion coherence (AQ group) * (dot lifetime) interactions with higher coherence threshold for limited dot lifetime stimuli is consistent with atypical magnocellular functioning, however psychophysical performance for those with High AQ is not explained fully, suggesting that other factors may be involved.

## Introduction

The autism spectrum is a heterogeneous class of disorders characterized by significant impairments in social interaction and communication, and the presence of restricted, repetitive and stereotyped behaviours [1]. In addition to these well-known higher order deficits in autism spectrum disorder (ASD), there also exists substantial evidence for atypicalities in lower level sensory processing, most notably, in the visual domain [2]. Although it remains unclear if an etiological role can be attributed to specific visual processing impairments, strong physiological evidence suggests a neurobiological origin [3] with a particular emphasis on the role of magnocellular and parvocellular physiological processes [4], the major inputs from geniculate to primary visual cortex (reviewed [5]). Three classes of perceptual abnormalities have repeatedly been associated with ASD, including superior processing of fine, local detail, inferior processing of overall global structure and impaired global motion detection (see [6] for a review). Evidence of a locally oriented visual processing system in ASD has been provided from studies demonstrating superior performance of visuospatial tasks compared to neurotypicals such as the Embedded Figures task [7], the Block Design task [8] and Navon Figures task [9]. Early studies have also identified a decreased ability to detect coherent motion [10–12]. However, the question of neural locus of motion processing abnormalities has been questioned following findings of normal flicker contrast sensitivity [13] and differences in second, but not first order motion perception [14,15].

The phenomena associated with perceptual abnormalities on visual tasks have been addressed by two hypotheses: the weak central coherence (WCC) [16] and the enhanced perceptual function hypothesis (EPF) [17]. The WCC hypothesis attributes autistic perceptual abnormalities to a deficit in global processing. The original WCC has however, since been modified with findings from a number of studies suggesting that autistics possess the ability to process globally when instructed to do so, however undergo preferential local processing when no such instructions are offered [18,19]. The EPF [17] takes a different stance, proposing a superiority of locally oriented processing in autistic individuals, without assuming a failure of global processing.

It was these initial observations that lead to investigations of early visual processing pathways, specifically the magnocellular and parvocellular streams in ASD. The magnocellular pathway and its cortical projections demonstrate greater sensitivity for moving stimuli, low spatial frequency, low contrast, and flicker and motion detection. Conversely, parvocellular inputs and their cortical projections have been implicated in colour perception, high contrast and fine form discrimination [20–25]. Single cell studies [26,27] of contrast response properties in primate lateral geniculate nucleus (LGN) show that magno-cells are characterized by high contrast gain and relatively small semi-saturation contrast in comparison to parvo-cells. In addition to differing contrast response properties, magnocellular and parvocellular pathways have been shown to have distinct latencies, with human VEP estimates indicating that the magnocellular pathway activates primary visual cortex (V1) 25–45 ms prior to parvocellular inputs [4,^28^,29]. This earlier arrival has been coined ‘the magnocellular advantage’ 30,31] – the earlier arrival of magno information to V1 is sufficient time for feedforward/feedback activation of the early dorsal cortical stream with feedback to Area V1 prior to the arrival of parvocellular afferent information into V1. Both cortical cooling of the dorsal stream [32,33] and lesion experiments in primate [34] point to a role for such feedback connections in aiding foreground/background segmentation in both single cell response properties and in perception.

*In-vivo* human electrophysiology, specifically visual evoked cortical potentials (VEPs), have been used to functionally isolate and directly examine differences in magnocellular and parvocellular function [29]. Non-linear VEPs (see Methods) capitalize on the different temporal response properties of the two streams, allowing delineation of separate contributions to the VEP [29] with a component of the second order kernel with high contrast gain, short latency and a saturating contrast response function being associated with magnocellular generation while another separate component with much lower contrast gain and without significant saturation, with a longer latency by some 25 ms, was identified as probably originating from parvocellular inputs. Such non-linear VEPs have been used to investigate the physiology underlying differences in autistic traits in neurotypical populations [4], with findings indicative of a magnocellular abnormality at high stimulus contrast in those high in self-rated autistic traits. However, the VEP peak contrast response functions of populations high, medium and low in autistic tendency have never been published.

Although limited in numbers, studies such as Sutherland and Crewther [4] are emerging that indicate that the perceptual abnormalities typical of ASD, such as impaired motion coherence [35] and local processing bias [36] may also occur in those exhibiting high autistic tendencies, but derived from the normal or typical (non-clinical) population. While the disorders which make up the autism spectrum have been diagnostically defined as discrete and internally homogenous, increasing awareness of the heritability of the disorder has led to a reconceptualization of the autism spectrum as existing within a broader autism phenotype. This suggests that milder autistic tendencies which echo the defining perceptual features of the autistic disorders, and even underlying physiology, may be continuously distributed throughout the general population. The Autism-Spectrum Quotient (AQ) [37] was designed to evaluate the extent to which an adult of normal intelligence exhibits characteristics associated with ASD. Generally, studies utilizing the AQ polarise their sample into comparisons of those low and high in autistic tendency, or in comparing those with ASD versus controls. Interestingly, although the development of the AQ was prompted by the recognition that autistic tendencies may exist on a continuum, research devoted to profiling autistic tendencies has failed to investigate the role of the midrange AQ scorers.

The current study thus aimed to investigate whether differences in degrees of autistic traits in young adults of normal intelligence would show differences in the contrast response functions of the impulse response (first order kernel) and second order Wiener kernels of the visual evoked responses to pseudo random luminance change. In addition, the physiological performance of the magnocellular and parvocellular visual pathways, derived from these measurements will be compared with psychophysical analysis of motion coherence discrimination at high and low contrast as well as under conditions more or less likely to require global motion assessment. As no prior research has been conducted on individuals scoring in the mid-range of autistic trait, the performance of the middle band should provide a reference for studies in vision, given differences observed across the “typical” population in terms of autistic tendency [4,6]. In addition, the presence of a middle band allows an assessment of whether autistic tendency is unimodal in terms of the physiological differences measured across groups.

## Methods

### Participants

216 participants were recruited through an online version of the Baron-Cohen et al., [37] AQ scale, within a study approved by the Swinburne Human Research Ethics Committee. The AQ was designed to investigate the degree to which adults of average intelligence (including those with clinical autism) have symptoms of the autism spectrum conditions. The questionnaire, well validated by control and clinical samples assesses five different areas relevant to the symptomology of the autism spectrum: social skill, attention switching, communication, imagination and attention to detail, with those scoring highly on the AQ tending to have more traits that are archetypal of a person with autism than those who have a low AQ score. Within our sample, AQ scores ranged from 3 to 37 with a mean score of 14.86 (*SD* = 6.22), which is comparable to previous normative data collected from control and student groups [38]. Low and High AQ group inclusion criteria were set at ±1 *SD* (6.22) of the mean. Thus, those scoring 8 or below (*n* = 31) or 22 and above (*n* = 27) met criteria for the Low and High AQ groups respectively. Participants scoring between 14 and 16 inclusive defined our narrow Mid AQ band (*n* = 36). Of the 94 individuals identified as belonging to Low, Mid or High AQ bands, 29 participants (15 male) were recruited for physiological and psychometric testing (see Table 1), giving written informed consent (according with the Declaration of Helsinki).

**Table 1:** Demographic details and AQ scores according to AQ group.

AQ Group	N	Mean AQ (SD)	AQ range	Mean Age, yr (SD)
Low	9	6.00 (1.32)	4–8	25.6 (5.0)
Mid	12	14.58 (0.79)	14–16	21.8 (1.9)
High	8	27.37 (5.13)	23–37	22.6 (1.2)

The use of a small range or AQ scores around the mean for the selection of Mid AQ group resulted in a clear separation of groups, as can be seen from the standard deviations in Table 1.

In order to assess non-verbal intelligence the original 36-item Raven's Advanced Progressive Matrices test [39,40] was administered with a time limit of 20 minutes. No differences were found between the Low, Mid and High AQ groups (see Table 2).

**Table 2:** Raven's Advanced Progressive Matrices scores (limited time) with means and standard error of the mean (SEM).

AQ_Group	N	Mean	Std. Error
Low	9	19.33	1.14
Mid	12	18.58	1.34
High	8	20.13	1.97

### Non-linear VEP

Non-linear achromatic multifocal visual evoked potentials (VEPs) were recorded from occipital cortex primary visual area (V1) using gold plated electrodes, placed at International 10/20 standard positions Oz and Fz, with an ear ground. Each participant in the Low, Mid and High AQ groups completed five VEPs of varying temporal luminance contrast (10%, 25%, 50%, 70% and 95%, with a mean screen luminance of 60 cd/m^2^) displayed using a binary pseudorandom stimulus sequence.

The stimulus, consisting of 19 hexagons of equal size, was presented using VERIS software (version 3.01 EDI, San Mateo, USA) on a 75 Hz frame rate monitor. Each of the19 hexagons fluctuated between two luminance levels using a pseudo-random binary m-sequence. This (m = 14) sequence, resulting in 2^14^ frames, changed at the frame rate of the monitor and thus the maximum temporal frequency was 75/2 = 37.5 Hz, while the lowest was approximately 2 Hz. The total sequence (at 75 Hz) took approximately 3.5 minutes. As the sequences are fully decorrelated, responses from all stimulus patches are available independently. For this experiment, only responses recorded from the central stimulus patch (subtending 5° of visual angle) were analyzed, due to the better signal to noise for foveal compared with peripheral patches, and to the variable efficacy of the generators in the folded cortical brain structure contributing to the VEP [4,29]. The signal was amplified 100,000 times with a notch filter at 50 hz, band-pass filtered between 3 Hz and 1 kHz and sampled at 1 kHz.

Kernels K1, K2.1 and K2.2 of the VEPs were extracted using VERIS 3.01 software via the Wiener kernel expansion [41,42]. The first order response (K1) is simply the sum of all responses to a white stimulus minus the sum of all responses to a black stimulus throughout the pseudorandom sequence (R_W_-R_B_). The second-order response takes account of the history of stimulation. The first slice of the second-order response (temporal non-linearity; K2.1) represents a comparison between two consecutive frames when a transition has occurred, and where a transition has not occurred (i.e. R_BB_+R_WW_−R_BW_−R_WB_). The second slice of the second-order response (K2.2) compares these responses with an additional intervening frame of either polarity. Thus, K2.1 measures neural recovery within the duration of one frame (13 ms with a 75 Hz frame rate) and K2.2 measures recovery over two frames (27 ms). The kernels separate inputs of the magnocellular and parvocellular pathways along the dimension of interaction time in slices of the second-order kernel [29] with K2.1 dominated by magnocellular input and K2.2 dominated by parvocellular input (on the basis of contrast gain, saturation and peak latency). After extraction, waveforms of kernels K1, K2.1 and K2.2 were analyzed using IGOR Pro 6.1 (Wavemetrics, Lake Oswego). Peak amplitudes and latencies in each kernel were compared across the three AQ sub-groups, at each of the five contrasts.

### Motion Coherence

A motion coherence stimulus with four conditions was constructed to investigate global and local function across the Low, Mid and High AQ groups. Using VPixx visual stimulation software (www.VPixx.com, version 2.72) a random dot kinematogram (in a circular window subtending 10°) comprising limited and unlimited lifetime dot conditions, was created. A proportion of the 300 dots was moving coherently in the same direction while the complement were moving in random directions at the same speed. Dot speed was one pixel per frame (2.65°/s) and each dot subtended 0.18°. In the limited dot condition, each dot would remain on the screen for just 6 frames (80 ms) then disappear and reappear in another location, maintaining the total number of dots on the screen (300) at any one time. In the infinite lifetime condition dots would traverse the stimulus with constant velocity from one side to the other. Stimuli were also presented at two different luminance contrasts – 24% and 95% (Michelson) around a mean luminance of 60 cd/m^2^ (measured using a **Red Tide USB650 spectrophotometer.** Ocean Optics, USA). For each condition there were four stimuli with dots (of variable coherence) moving up, down, left or right. During the task, participants were asked to select the direction of the coherent motion under 4 alternate forced choice conditions (up, down, left or right). The 2×2 (Dot Lifetime×Contrast) design was utilized to account for possible group differences in the ability to track individual dots (which the limited lifetime conditions prevented) as well as two different luminance contrasts, designed to alter the balance of magno and parvocellular contribution to the coherent motion assessment.

Thresholds were estimated via the inbuilt VPEST routine in VPixx which fits the total data trial-by-trial (Weibull fit function with shape parameter beta = 3), producing an estimate of threshold which is then used as the stimulus level (percentage of the dots moving coherently) for the next trial. Fixed value replication trials (5) were used to ensure the threshold region was not reached too rapidly, stabilizing the threshold estimation. At least 30 trials for each of the four thresholds were completed. Following outlier removal (data lying more than 2 SD from their mean), thresholds were statistically compared between groups and condition using SPSS 19.0 software (IBM, New York, USA) and jmp v10 (SAS Corporation).

## Results

### Non-linear VEP

**Figure 1:**
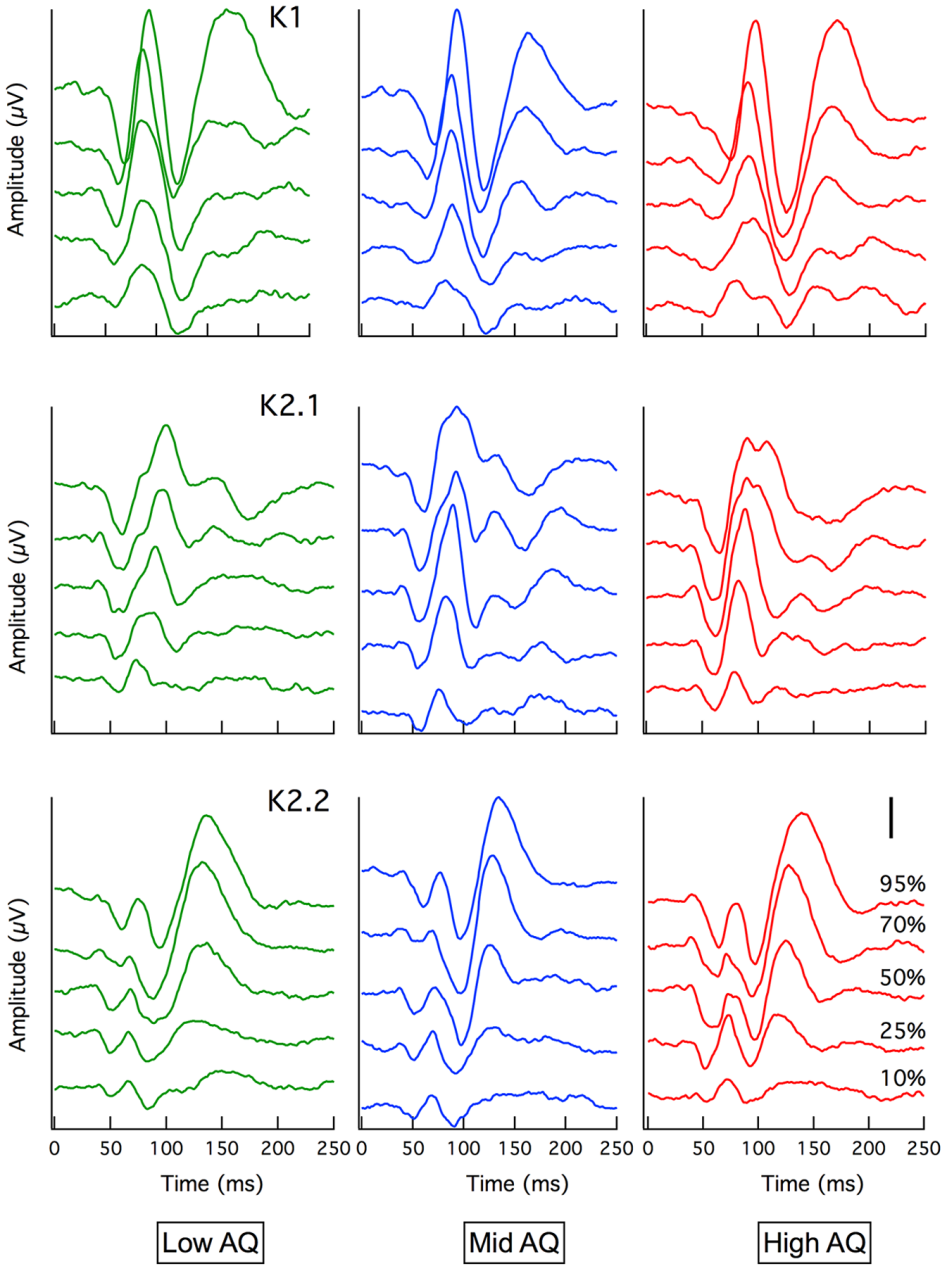
Grand mean average VEP. Waveforms are shown for Low (green), Mid (blue) and High AQ (red) for K1, K2.1 and K2.2 kernels. Separate traces represent evoked potentials to different temporal luminance contrast from 10% (bottom trace) to 95% (top trace). Larger amplitudes with steeper gradients can clearly be seen in the K2.1 waveform for the High AQ group compared with Low AQ. Note: Scale bar = 500 nV

The cortically recorded VEP first order response (K1) showed the expected variation in amplitude with contrast [29]. Initial departure from baseline occurred at around 45 ms. Mean average amplitudes and latencies were compared between Low, Mid and High AQ groups for the first-order peaks (K1: N60P90, N125P160), first slice of the second order response (K2.1: N60P90) and second slice of the second order response (K2.2: N60P75, N95P130) at each of the stimulus contrasts recorded (10%, 25%, 50%, 70%, 95%), (see Figure 1). Some departures from the data of Klistorner et al. [29] (recorded at a slower stimulus frame rate) are apparent. The first is the emergence of a second positivity in the K1 waveforms of all groups in the current recordings at long latencies (around 175 ms). The second is the appearance at short latencies in the second slice K2.2 waves of a small N60P75 complex. This was not observed in the original study [29].

The waveforms of the first order (K1) peaks showed little qualitative difference between groups, with only the P1 latency showing a perceptible delay at the highest contrast level for the high AQ group, the mean latency greater by approximately 6 ms (High AQ 97.8±3.9 ms; Mid AQ 92.1±5.5 ms; Low AQ 92.3±5.5 ms).

The second order kernel responses showed that the High AQ first slice response (K2.1) developed a second (or extended) positivity at high contrast, rather like that reported by Sutherland and Crewther[4]. Here we note that larger amplitude second order responses are indicative of poorer recovery after stimulation (thus, a very rapid photosensitive device such as a phototransistor demonstrates zero second order responses when stimulated at the rates used in this experiment).

**Figure 2:**
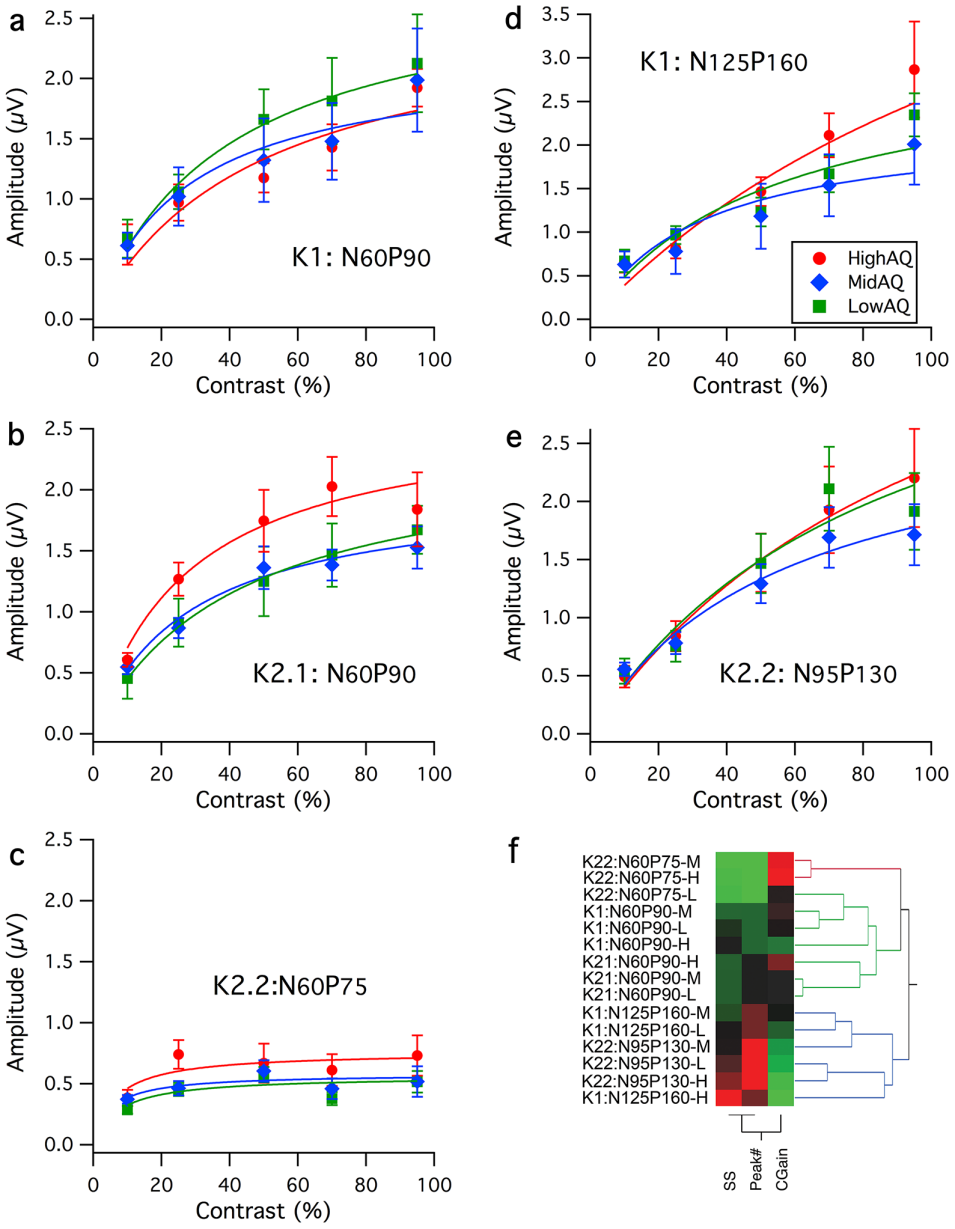
Contrast response functions for five major peaks. Contrast response functions for High (red), Mid (blue) and Low (green) AQ groups for major peaks of the first order (K1) and first two slices of the second order (K2.1, K2.2) kernels. Mean amplitudes (with SE) are shown with Naka-Rushton fits for **a**: The initial N60P90 peak of the first order kernel K1, **b**: The N60P90 peak of the second order kernel – first slice K2.1, **c**: the small N60P75 peak of the second order kernel – second slice K2.2 **d**: the longer latency N125P160 peak for the first order kernel K1, **e**: the major N95P130 for K2.2. **f**: Hierarchical decomposition of the 15 contrast response functions on the basis of Semi-Saturation constant, Contrast Gain, and Peak Number shows a strong separation of waves previously attributed to magno and parvocellular origin.

Contrast response functions are plotted as response amplitude versus contrast for the five major peaks visible in Figure 1. These comprise in first order responses, the N60P90 and N125P160 peaks, for the K2.1 kernel slice the N60P90 peak and for the K2.2 kernel slice the diminutive N60P75 peak and the much larger N95P130 peak. Mean amplitudes with standard errors are shown in Figure 2. The gain and semi-saturation characteristics of the major peaks were estimated through Naka-Rushton fits (see Figure 2), employing the equation: 
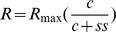
 where the response 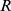 as a function of contrast 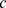 is described by a maximum value 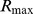 and a semi-saturation value 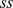. Qualitatively these show characteristics as described by Klistorner et al – the putative magnocellular nonlinearity (N60P90 dominating the K2.1 response) showing high contrast gain and relatively strong saturation with contrast, while the putative parvocellular nonlinearity (longer latency N95P130 dominating the K2.2 response) shows smaller contrast gain and much less evidence of saturation at high contrast. Also, it is clear that the High AQ group shows differences from the other two autistic tendency groups for some, but not others of the peaks. The fit parameters of Contrast Gain, Semi-Saturation and Peak number were fed into a hierarchical decomposition analysis tool (jmp software, v10, SAS Corporation). The tool segregated the early waves for K1, K2.1 and K2.2 from the behaviours of the other peaks.

The detailed values of Semi-Saturation contrast and Contrast Gain (slope of the Naka-Rushton fit at zero contrast) for each of the five major peaks are shown in Table 3. The uppermost two rows of Table 3 comprise the peaks identified by Klistorner et al [29], the major peaks of the first and second slice K2.1 and K2.2. The third row represents the parameters obtained for the small amplitude short latency K2.2 peak. As its amplitude is much smaller than the later N95P130 peak (which continues to grow at high contrast), there is likely to be an impact on the estimated semi-saturation constant, due to potential cancellation of the P90 and N125 components.

**Table 3:** Naka-Rushton fit parameters.

		High AQ		Mid AQ		Low AQ	
Kernel	Peak	SS%	CG	SS%	CG	SS%	CG
K2.1	N60P90	27.7	9.6	28.2	7.1	28.2	7.1
K2.2	N95P130	112.0	4.3	56.9	5.0	86.9	4.7
K2.2	N60P75	6.6	11.7	5.1	11.3	7.4	7.7
K1	N60P90	48.3	5.4	27.0	8.1	38.6	7.4
K1	N125P160	161.1	4.2	32.1	7.0	53.4	5.8

Semi-Saturation contrast (SS%) and Contrast Gain parameters (CG) extracted from Naka-Rushton curve fits.

### Statistical Differences between AQ groups

Statistical comparison of the data was carried out using a mixed design repeated measures general linear model (5 Contrast×3 AQ Groups). Within subject contrasts showed the expected strong effect of stimulus contrast for most peaks.

#### First Order

The greatest difference between AQ groups was observed in the K1: N125P160 peak. Qualitatively, Figure 2c shows that the amplitude is a non-saturated function of contrast, which appears measurably larger for the High AQ group for all contrasts measured greater than about 20%. Statistically, this was confirmed with a generalized linear model fit showing significant difference between groups (Chi-square = 7.74, *p* = .02).

#### Second Order, First Slice

Following previous analyses, the major waveform characterized by the K2.1: N60P90 amplitude was analyzed in a similar fashion (generalized linear model fit), and the AQ groups showed significant difference (Chi-square = 9.88, *p* = .007). Contrast tests showed the Low and Mid AQ groups to be insignificantly different while the High AQ group showed greater non-linear amplitude.

#### Second Order, Second Slice

The early K2.2: N60P75 wave showed a rapidly saturating form with the High AQ group showing greater amplitude. The groups were again significantly different (Chi-square = 10.4, *p*<.006), but again the difference between Low and Mid AQ groups was insignificant. The later K2.2: N95P130 wave showed a quite linear increase in amplitude with contrast and little evidence of saturation. There was no significant difference between groups (Chi-square = 1.92, *p* = .383).

**Figure 3:**
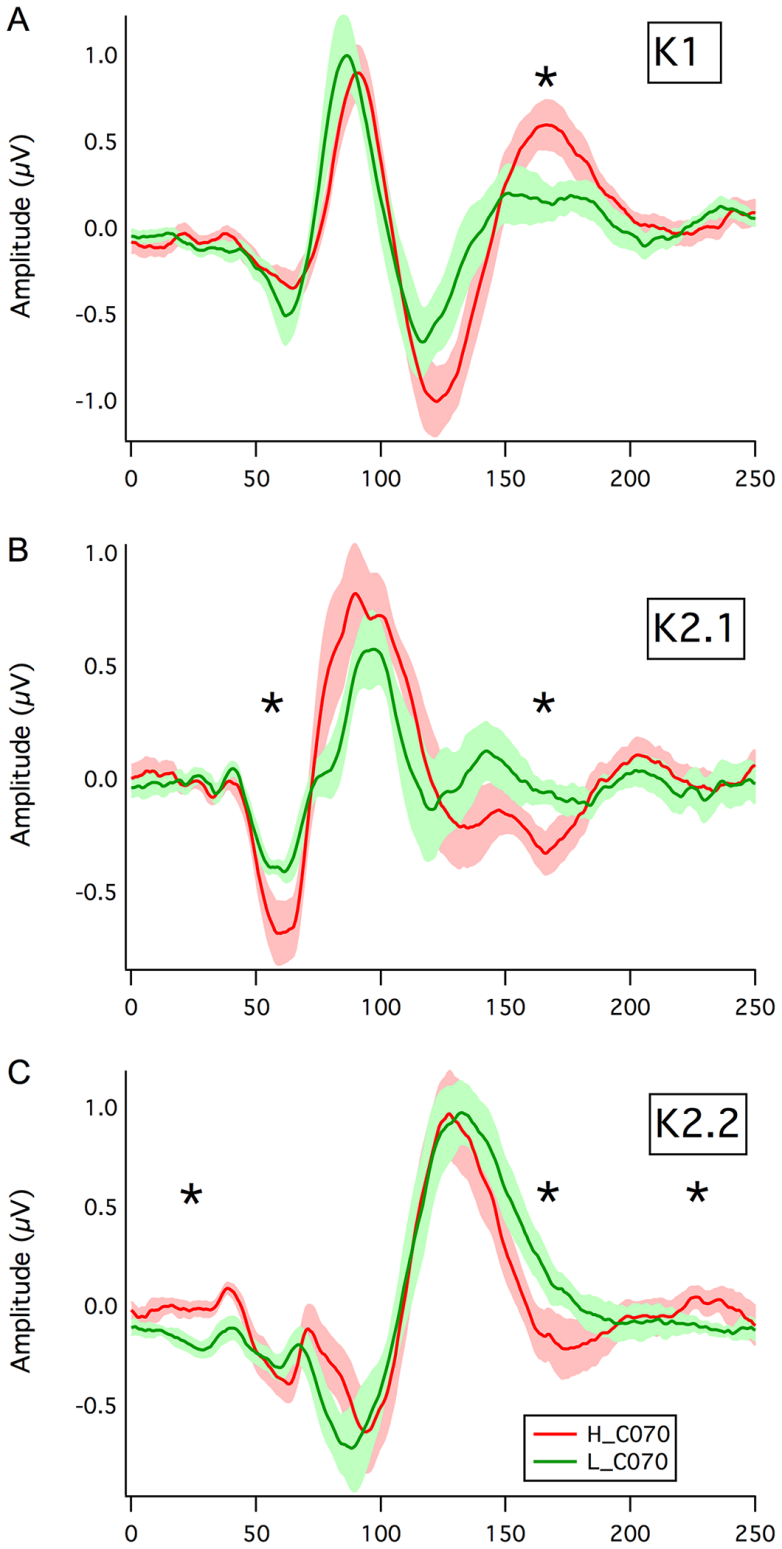
Waveform Comparison. Comparison of the grand mean average waveforms for **a**: K1, **b**: K2.1 and **c**: K2.2 kernel responses recorded at 70% stimulus contrast. The mean waves for the High and Low AQ groups are shown with a fringe of ±1SE. Significant mean waveform departures (*p*<.05) are indicated by asterisks.

The grand mean wave averages were also inspected for differences between High and Low AQ groups (see Figure 3). For illustration, grand mean averages for High and Low AQ groups for the 70% contrast recordings were plotted together with fringe values equal to one standard error of the mean, calculated on a point-by-point basis using Welch's t-test [43] (for unequal sample size without assumption of equal variance). For the first order kernel, deviations did not occur until around 120 ms with the evoked response for the high AQ group significantly greater around 160 ms than for the Low AQ group. Several differences between High and Low AQ groups emerged for the second order responses. For K2.1, the non-linearity generated by the High AQ group was significantly greater at around 60 ms latency. While the P90 positivity was much more robust for high *cf* low AQ, the mean difference did not achieve significance. A small departure at around 160 ms was also significant. For the K2.2 second slice, a very early difference was apparent over latencies 20–40 ms. In addition, the High AQ mean wave recovered to baseline more rapidly than Low AQ resulting in a significant difference at around 160 ms.

### Motion Coherence

**Figure 4:**
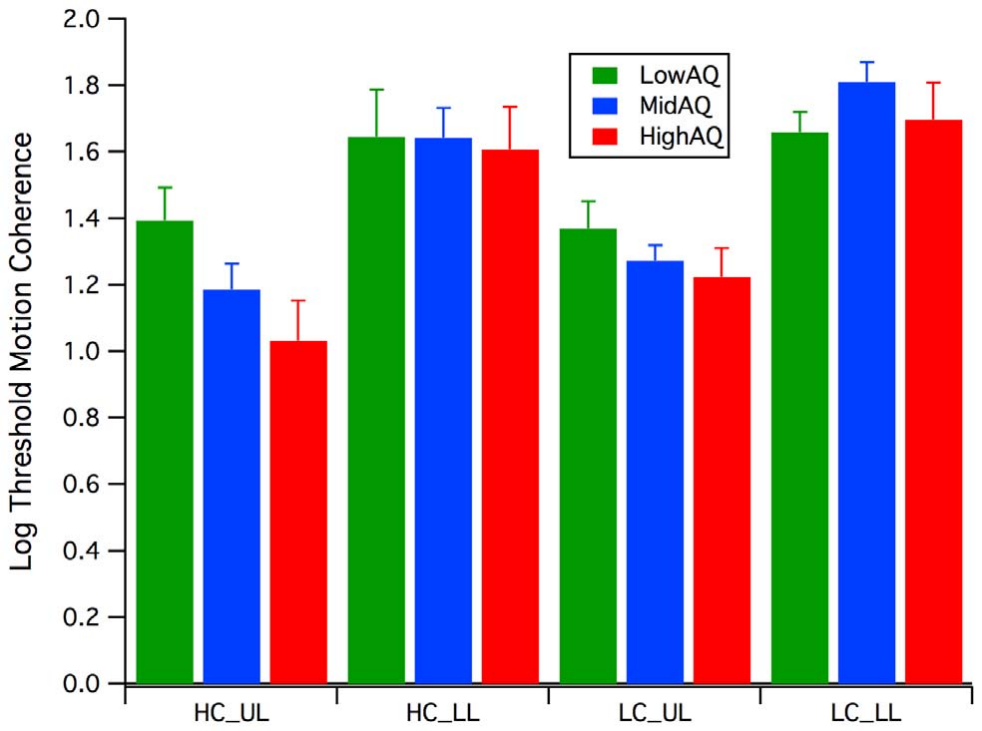
Motion Psychophysics. Mean log percent coherence thresholds for Low (green), Mid (blue) and High (red) AQ groups across the two contrast conditions (High Contrast HC, Low Contrast LC) and two lifetime conditions (Unlimited Lifetime UL, Limited Lifetime LL).

Inspection of the mean log motion coherence thresholds (see Figure 4) shows a manifest effect of Dot Lifetime, with the Limited Lifetime (LL) thresholds being much greater than those when the dots maintained a constant path across the screen (Unlimited Lifetime - UL), with analysis of variance (ANOVA) on the log thresholds revealing a significant main effect of Dot Lifetime (*F*(1, 27) = 67.8, *p*<.0005). A 2 (Dot Lifetime: LL, UL)×2 (Contrast: HC, LC) between groups repeated measures analysis revealed a significant Dot Lifetime * Group interaction (*F*(2, 25) = 3.4, *p* = .05), while there was a marginal main effect of contrast *F*(1, 25) = 3.3, *p* = .08).

A similar pattern of motion coherence results was reported by Sutherland and Crewther (2010)[4]. The large variance in the limited lifetime conditions, especially at low contrast, where threshold was approaching ceiling level for some participants may have impacted on sensitivity.

## Discussion

Mechanisms underpinning ASD remain highly controversial with opposing theories arguing Autism to be a result of either a top-down ‘social orienting’ deficit [16,44], or as a result of bottom-up deficits [17,18]. The results of the current study suggest that even in a sub-clinical high autistic tendency group, early visual processing abnormalities are present. Interestingly, although aberrant magnocellular pathway activation was clearly recorded in the high AQ group, with an abnormally high level of nonlinearity in magnocellular evoked responses, the relationship to the low-level perceptual tasks coherent motion analysis requires discussion. The significant interaction found between Dot Lifetime and AQ group is perhaps best interpreted as the High AQ group being less able to perform well when forced to the more global percept of dots whose lifetime on the screen was only 80 ms. That is, the possibility of tracking individual dots in order to make a direction judgement was removed. Hyperperformance in single dot tracking mode (unlimited lifetime) is perhaps less surprising given claims of hyperacuity in autism [45] using similar adaptive thresholding paradigms This has been suggested to relate to lower random error rates in the High AQ group compared with the other groups [46], with modeling showing adaptive staircase paradigms to be vulnerable to such differences [47]. Also, direct measures of acuity [48] and of contrast sensitivity across a range of spatial frequencies [49] have failed to replicate such acuity differences.

The larger, non-saturating first order (K1) response seen in the N125P160 mean wave for the High versus Low AQ group (Figure 3a), together with the more rapid recovery in the K2.2 kernel response around 160 ms latency (Figure 3c) also gives evidence for a stronger occipital activation for the High AQ group compared with Low AQ. In addition, while both magno and parvocellular afferents are likely to contribute, given the long latency, the lack of saturation suggests that at least at high contrast such responses are dominated by the non-saturating parvocellular input. Such higher V1/V2 activation is supportive to a degree of the EPF hypothesis of autistic perception.

Non-linear multifocal VEPs allowed for the simultaneous recording and subsequent delineation of separate contributions of magnocellular and parvocellular inputs to the visual evoked response recorded from visual cortex V1. In addition to the initial characterization by past studies [29], contrast response functions recorded here further supported the separate contributions of magnocellular pathways to the second-order response first slice (K2.1) and parvocellular inputs to the main wave complex of the second-order response second slice. However this major K2.2 disturbance was preceded by a smaller N60P75 contribution, not reported by Klistorner et al. [29]. We would argue that this wave is generated by the magnocellular stream, on the basis of the saturation of the contrast response shown in Fig 2c (with a semi-saturation contrast of <20%), higher amplitudes for the high AQ group compared with the other two groups (similar to that observed with the K2.1 major non-linearity), and also the very similar latencies of the K2.2 N1 P1 complex and the N1P1 complex in the K2.1 response. This magnocellular contribution to the second slice was observed here, but not in the original study [29] This is probably due to the higher frame rate of stimulation used here (75 Hz) *cf* the Klistorner study (67 Hz).

The claim for isolation of magno and parvocellular generated contributions to the cortical nonlinear VEP requires comparison with primate recordings from the LGN where there can be no doubt of the source of responses. In comparing the cortical VEP and single cell LGN data (see Table 4), it should be realized that different, unrelated units are employed. Also, the VEP is a massed response, while the LGN data is of impulse rates from single cells.

**Table 4:** Comparison of Single cell and VEP Contrast Gain and SemiSaturation parameters*.

Nonlinear VEP Kernel	Wave	Contrast Gain (μV)	SemiSaturation %
K2.1	N60P90	7.4	32.7%
K2.2	N95P150	4.6	85.2%

Single Cell	Cell Type	Contrast Gain (Spikes/s)	SemiSaturation %
RGC	Magnocellular	538.6	12.36%
RGC	Parvocellular	48.9	56.3%

* Single cell (LGN) data fitted from Kaplan and Shapley [26], while VEP data are mean values from Table 3.

At first glance, there does not appear to a strong similarity between the primate geniculate data and human cortical VEP – for example, the units of Contrast Gain are totally different. However, more importantly, the VEP data represents the cumulative effect of thousands of neurons, and it is estimated that the population of parvo-cells is approximately 8 times that of magno-cells [50,51]. A simple linear superposition would then estimate the contrast gain for the VEP to be relatively 8 times greater for the parvo compared with the magno generated responses. The contributions of cortical neurons driven by magno and parvo afferents should also take into account the relative rectification of firing in these neurons, suggesting the possibility of high relative recruitment as a function of contrast. This could help to explain qualitatively the observation that the semi-saturation constants of both putative magno and parvo generated non-linearities are greater than the LGN measurements.

Visibly greater amplitudes in magnocellular nonlinearities in the high AQ group suggest poorer recovery of the magnocellular system in the high AQ group during rapid stimulation. This feature was reported by Sutherland and Crewther [4], who reported a delay in recovery of the major positivity of the first slice of the second order kernel response. A similar delay was also visible in the High AQ group K2.1 responses at high contrast (see Fig. 1). Similar responses between AQ groups for the N95P130 peak of the K2.2 nonlinearity (Figure 2e) suggest no early visual parvocellular abnormality in autistic tendency. The difference observed in the N125P160 peak amplitude for the K1 mean average waves between High and Low AQ groups (Fig 3a) is sufficiently late that it could reflect contributions both from magno- and parvocellular streams, as well as feedback from higher cortical areas.

Inefficient recovery in the magnocellular pathway – the system that dominates the input to motion sensitive areas of the brain, might predict poor coherent-motion detection in autistic groups [10,^12^,13] due to visual information not being sampled at a sufficiently rapid rate. However in the unlimited lifetime condition the High AQ group showed an enhanced ability to locally track individual dots within the motion coherence array. This may be an example of enhanced low level perceptual functioning, as outlined by the EPF hypothesis [18]. Despite performing the best of the three groups in the high contrast, unlimited lifetime condition, the High AQ group failed to outperform in the limited life conditions. In this condition, the ability to locally track dots was inhibited by restricted dot lifetime, participants being required to integrate global processing to a greater extent. The current study provides evidence for mechanisms that might underlie such failure, suggesting problems in coping with rapid visual stimulation, though not in sensing collective motion. In addition, the slow drift rate employed (1 pixel per frame equates to 2.5°/s angular velocity when viewed at 57 cm) could be coded easily by parvocellular neurons [23]. Marginally increased magnocellular latencies and poor magnocellular recovery, as indicated by the larger amplitudes of the K2.1 major peak, reported in the current study may result in a decreased ability to integrate contextual information into low-level visual inputs. Also, the superior amplitudes of late physiological K1 responses recorded in the High AQ group, may equate to increased visual sensitivity, at least in occipital cortex. Other studies, eg, Bertone et al.[14] found no difference between autistic individuals and controls for luminance-defined motion.

In addition to examining the visual processing of High versus Low AQ groups, an additional aim of the current study was to investigate the poorly defined AQ middle band. Based on the current trends within the literature it was expected that the Low, Mid and High AQ groups would demonstrate progressive differences, thus supporting the idea of autistic tendencies existing along a continuum [52]. While significant differences were evidenced between the Low, Mid and High AQ groups, a clear and defined profile for the Mid AQ group was not displayed in the VEP or motion coherence tasks.

While the current study investigated early visual processing in typical populations, in light of the mounting evidence for a continuum of symptoms and severity in ASD, there is no reason that the results of the current study cannot be generalized to profiles of high functioning autism or Asperger's syndrome. Indeed in recent imaging studies of clinically defined autism [53] the AQ scores of the ASD group were as low as 18, with a mean value of 29 – a similar value as the mean score (27) of the High AQ group in the current study. While a magnocellular deficit theory appears a plausible means of explaining the atypicalities of visual perception in ASD [6], the degree to which these underlying processing and perceptual mechanisms present during development may help to explain common behavioural deficits of autism remains a matter of speculation. The fact that the magnocellular-derived physiology of the High AQ group was distinct from that of the Mid and Low AQ groups, but that Mid and Low AQ groups performed very similarly, suggests a unidimensional nature of autistic tendency as represented by the AQ score. The lower mean log unlimited dot lifetime thresholds for the High AQ group compared with the other two groups suggests that part of the performance measure may derive from single dot tracking, with the High AQ groups showing an enhanced ability to track single dots to interpret motion direction. Thus performance on random dot tasks may involve a combination of hyperattention (for single dots) combined with impaired global motion capability.

In conclusion, this study has demonstrated pathway specific abnormalities for those high in autistic traits in a fashion that could help to bring together theoretical models of autism. The larger contrast response functions exhibited by the High AQ group for the first slice second order components indicates a less efficient use of the magnocellular dominated dorsal system as it reflects less preparedness to fire again under conditions of high temporal frequency stimulation. Given the strong connection between magnocellular afferents to cortex and the dorsal cortical stream, connections with impaired figure-ground perception and spatial attention are possible. These latter properties may contribute to an impaired global perception, as postulated by the WCC hypothesis. This also is likely to explain the more local visual perception demonstrated by those high in autistic tendency, except when top-down global attentional selection is mandated. The EPF hypothesis also gained some support from the larger amplitude first order long latency waves recorded for the High AQ group indicative of enhanced occipital activity, the lack of saturation with contrast indicating a dominant role of parvocellular processing in this evoked potential.
